# Richard Hugh Brew

**Published:** 1917-07

**Authors:** 


### Obituary.

### RICHARD HUGH BREW.

DURING the twenty-three years that Richard Hugh Brew practised at Chew Magna he enriched the life of the neighbourhood, not only by his sound medical judgment and keen enthusiasm for his work and his patients' welfare, but by his exceptionally manly, amiable, yet forceful disposition ; thus his death at the age of 55 leaves a gap that none can fill for many a year.

He was born at Ballyvaughan Rectory, Co. Clare, the only son of Richard St. John Brew, an officer who died at the early age of 27, and was educated at the medical schools of Dublin and at Edinburgh, where in 1884 he obtained his diplomas of L.R.C.P., L.R.C.S., and L.M. Edin. After a short assistantship at Chesham, he practised in Birmingham, but moved to Chew Magna shortly after his marriage in 1890.

His many friends and admirers in Bristol will realise how true is the pen-portrait of Brew by his friend, written for the *British Medical Journal*, as follows :—

"Of manly physique, the picture of health and vigour, his bodily presence was the outward sign of the fine, strong spirit within." The writer (H. W. P.) had known him for the last eleven years, and as "brother, sometimes patient, and friend had come to hold him, from his sterling worth of heart and head and his sound professional knowledge, in the highest esteem. Gifted in no ordinary degree with the power of intuition, which is really the result of deep-laid and thoughtful experience, he went quickly and surely to the root of every malady, and seemed to have a right judgment in all things. Prompt in decision, fearless in opinion, without hesitation or ambiguity of speech, he straightway inspired confidence ; and take him for all in all, he was to my mind one of the best doctors I have ever known. Then, alas ! in the full tide of his work and prosperity, he was smitten down in the spring of 1915 by an obscure illness, due, it was thought, to some bacillary infection. He was at death's door, but rallied, recovered—it was thought permanently—and had been at work again for nearly a year, when there was a relapse in severer form. Nor can it be doubted that stress of work, the grave anxieties of this disastrous time, and, above all, the loss of Cyril, his much-loved younger son—a charming, gallant boy, who held a commission in the Irish Guards, and who died somewhere near Ypres after terrible wounds last October—undermined his natural strength and lowered his resisting powers."

"Only a country practitioner, but withal a man of noble, stainless character, of true humility and 'gentleness untired,' a keen sportsman, ever ready to befriend the poor, and implicitly trusted in every walk in life."

